# Effects of the COVID-19 Lockdown on Portuguese Children’s Motor Competence

**DOI:** 10.3390/children8030199

**Published:** 2021-03-07

**Authors:** André Pombo, Carlos Luz, Cristina de Sá, Luis Paulo Rodrigues, Rita Cordovil

**Affiliations:** 1 Faculdade de Motricidade Humana, Universidade de Lisboa, 1499-002 Cruz-Quebrada, Portugal; 2 Escola Superior de Educação de Lisboa, Instituto Politécnico de Lisboa, 1549-003 Lisboa, Portugal; carlosl@eselx.ipl.pt; 3 Centro Interdisciplinar de Estudos Educacionais, CIED, 1549-003 Lisboa, Portugal; 4 Departamento de Ciências do Movimento Humano, Universidade Federal de São Paulo, Santos 11015-020, Brazil; cristina.sa@unifesp.br; 5 Escola Superior de Desporto e Lazer, Instituto Politécnico de Viana do Castelo, 4960-320 Melgaço, Portugal; lprodrigues@esdl.ipvc.pt; 6 Research Center in Sports Sciences Health Sciences and Human Development, CIDESD, 5000-801 Vila Real, Portugal; 7 CIPER, Faculdade de Motricidade Humana, Universidade de Lisboa, 1499-002 Cruz-Quebrada, Portugal; cordovil.rita@gmail.com

**Keywords:** lockdown, COVID-19, motor competence, physical activity, children

## Abstract

During long periods without school, children are more susceptible to unhealthy behaviors, such as an increase in sedentary behaviors, which has a negative impact on children’s motor competence (MC). The COVID-19 lockdown offered us a unique opportunity to test, in a quasi-experimental setting, the impact of lockdown movement restrictions on children’s MC. We assessed the motor competence of 114 children aged 6–9 years using the motor competence assessment. All children were tested before and after the COVID-19 lockdown. Chi-square and 2 × 2 ANOVA (sex by moment) were used to further analyze the data. Regardless of sex, motor performances in all tests (except for jumping sideways in boys) were lower when compared with performances before lockdown. There was a marked decreasing trend in children’s levels of MC, shifting from an upper to a lower quartile in different tests. The results after the lockdown were always significantly inferior to the results before lockdown in all motor tests (except jumping sideways), in the three components of MC, and in global MC. Children’s global MC score decreased by an average of 13 points in boys and 16 points in girls. The imposed movement restrictions had a negative effect on children’s motor competence development.

## 1. Introduction

Motor competence (MC) is a person’s ability to be proficient in a broad range of locomotor, stability, and manipulative skills [1,2]. This proficiency relates to the development and performance of human movement in a range of fundamental movement skills (e.g., throwing, catching, running) [3] and shapes the foundation for developing more specialized movements sequences [4], which may lead to lifelong physical activity (PA) and movements skills [5].

The theoretical model proposed by Stodden and colleagues (2008), and corroborated by Robinson and colleagues (2015), suggests that MC is a key role in human development—low levels of MC during childhood could compromise the adoption of active and healthy lifestyles [6,7]. Children with low levels of gross MC tend to be less physically active and have lower levels of cardiorespiratory fitness [8]. On the contrary, higher MC attenuates the decline in PA levels throughout childhood [9] and is associated with higher levels of PA and fitness in adolescence [10,11]. This relationship between MC and PA has been postulated as bidirectional, depending on the child’s developmental stage [7,12,13]. In fact, childhood physical inactivity is associated with difficulties in developing appropriate motor competence levels [14].

In recent years, with the social and technological changes, we observed adverse impacts for children’s development, especially regarding PA [15]. Nowadays, children spend more time in sedentary activities and less in physically active ones [16,17] when compared to past generations [18]. Motor skill development and physical fitness show a secular decline [19,20], which negatively impacts various health outcomes [21].

Since the outbreak of the COVID-19 pandemic, many aspects of children’s daily lives have been disrupted. All over the world, governments have imposed measures of social distance, closing schools, and sports clubs, depriving children of most kinds of movement experiences. In Portugal, the entire school system closed on March 16th and was transferred to a mixed system of broadcast television and online homeschooling from April to June. All sports and physical activities were suspended until September, and leisure activities were also strongly restricted by the measures imposed by the lockdown. These measures led to a long period of movement restrictions in children’s lives. Confined to their homes, without any organized PA, free playtime outdoors, or opportunities to play with friends, children decreased their PA behaviors [22,23,24,25], increased screen time [23,24,26], and changed their eating habits, in most cases for worst [25,27,28]. These changes in children’s routines, as well as the correlates that influenced them, were investigated by survey studies during lockdown [22,23,24,25,29] because there was practically no other way of collecting data during that period. However, the objectively measured impact of these restrictions on Portuguese children’s motor behavior is yet to be determined, since, to our knowledge, no previous study has compared motor assessments pre- and post-lockdown.

Knowing that in Portugal, school physical education (PE) is mandatory (from 90 to 150 min/per week), that school recesses are usually a time for students to be active [30]; and that almost half of the Portuguese children are enrolled in some sort of sports club [31], we have to recognize that schools and sports clubs have a major role in promoting PA habits in this population. 

We know from previous studies that during long periods without school, children are more susceptible to unhealthy behaviors, such as increased sedentary behavior [32,33] and this has a negative impact on children’s MC [34], as well as on their body composition and cardiovascular fitness [35]. Given the ethical implications, imposing children to long periods without movement to test for the effect of PA restriction was never an option. The COVID-19 lockdown (unfortunately) offered us a unique opportunity to test this in a quasi-experimental setting, by forcing all children to stay home with severe movement restrictions for a long period of time. What are the impacts of the lockdown on children regarding their motor development? 

With this study, we aim to examine the impact of the COVID-19 lockdown on children’s MC using a standardized assessment protocol. It was hypothesized that the imposed movement restriction had a negative effect on the development of children’s motor competence.

## 2. Materials and Methods

Prior to the lockdown, we had recently completed the MC assessment of a group of children for an intervention and follow-up study that had to be postponed due to the circumstances. However, these assessments made it possible to compare the results of the same children after the lockdown, enabling us to determine the effect of the movement restriction on motor competence development.

### 2.1. Participants

Before the lockdown, we had assessed the motor competence of 182 children. At the start of the new school year, the sample was reduced to 114 children (50 boys and 64 girls; mean age of 7 years old) because some of the children previously assessed had since progressed into middle school.

Children were selected from a public school in the Lisbon district and had no motor, cognitive, or health impairments (parent reported) that could affect their performance on the motor tests. Three trained physical education teachers collected the data at both time points. The ethical committee of the Lisbon School of Health Technology approved the study procedures regarding scientific research involving human subjects (CE-ESTeSL-N°. 47-2019). Written informed consent was obtained from the school director and the parents of all participants. Verbal assent was obtained from the children before data collection.

### 2.2. Measures

MC was evaluated with a valid quantitative instrument, the motor competence assessment (MCA) developed by Luz and colleagues [2]. This instrument is composed of two tests for each MC subscale (stability, locomotor, and manipulative). Stability tests: Shifting platforms required subjects to move sideways for 20 s using two wooden platforms (25 cm × 25 cm × 2 cm with four 3.7 cm feet at the corners). Each successful transfer from one platform to the other is scored with two points (one point for moving the platform; one point for moving into the platform). Participants completed two trials and the best score was recorded. Lateral Jumps required subjects to jump sideways with two feet together over a small wooden beam (60 cm length × 4 cm high × 2 cm width) located in the middle of a rectangular surface (100 cm length × 60 cm width) as fast as possible for 15 s. Each correct jump (two feet together, without touching outside the rectangle, and without stepping in the wooden beam) was scored 1 point and the best score was recorded. Locomotor tests: The shuttle run (SHR) required subjects to run at a maximal speed between the starting line and a line placed 10 m away. Beginning at the starting line, subjects ran to the opposite line, picked up a block of wood, ran back, and placed the block beyond the starting line. Subjects then ran back to retrieve the second block and carry it back across the starting line to finish the test. The best time in seconds of the two trials was recorded. The standing long jump (SLJ) required subjects to jump with both feet simultaneously as far as possible. The best score of 3 attempts was the longest distance in cm between the starting line and the back of the heel at landing. Manipulative tests: Throwing velocity required subjects to throw a ball against a wall at maximum speed using an overarm action with a preparatory balance (one or two steps). For children between 7 and 10 years old, a tennis ball was used (diameter: 6.5 cm; weight: 57 g). For children 11 years old and older, a baseball was used (diameter: 7.3 cm; weight: 142 g). Peak velocity was measured in m/s with a velocity radar gun (Pro II Stalker radar gun). Every participant performed three trials, with the final score being the best result. Kicking Velocity required subjects to kick a soccer ball against a wall at maximum speed using a preparatory balance (one or two steps). For 7- and 8-year-old children, a soccer ball n° 3 was used (circumference: 62 cm, weight: 350 g). For 9- and 10-year-old children, a soccer ball n° 4 was used (circumference: 64 cm, weight: 360 g). For subjects older than 10 years of, a soccer ball n° 5 (circumference: 68 cm, weight: 410 g) was used. Ball peak velocity was measured in m/s with a velocity radar gun (Pro Stalker II radar gun). Every subject performed three trials, with the final score being the best result.

### 2.3. Procedures

Two testing sessions, one before (December 2019) and one after lockdown (September 2020) were used. For all MC tests, three experienced researchers followed the respective test protocol. All participants completed a 10 min general and standardized warm up before the beginning of the tests. After a proficient demonstration of each test technique with verbal explanation, participants were allowed to try each test once before being assessed. Children performed all the tests in small groups (usually approximately 5 children for each task). Motivational feedback was provided by the researchers, but verbal feedback on skill performance was not, as advised in the test protocol [2]. The same procedures were used for all tests.

### 2.4. Statistics

The raw results of all tests were transformed to percentile values, according to the Portuguese MCA norms [3]. MCA subscales scores were calculated as the average of the percentile values of the corresponding two motor tests, and total MCA scores were calculated as the average of the three subscales. 

To analyze the possible change in percentile distribution (adjusted to age and sex) the subjects were classified for each test at each time point (before and after lockdown) into four groups according to their percentile score (Q1 < p25; 25 < Q2 < p50; 50 < Q3 < p75; and Q4 > p75). Chi-square McNemar–Bowker Test was used to test for differences in the distribution between the two time points.

A repeated 2 × 2 ANOVA (sex by time) was conducted to determine the effect of sex (boys and girls), time (before and after lockdown), and interaction (sex × time) for each motor test, MCA subscales, and total MCA. Kolmogorov–Smirnov test was used to identify normality, and all assumptions for repeated one-tailed ANOVA were met. The power of the McNemar test was 0.70, calculated from the sample size of this study (n = 114) by GPower 3.1 (Macintosh, Dusseldorf, Germany), types of analysis compromised. The Statistical Package for Social Sciences (SPSS) for Macintosh, version 25.0, Armonk, NY: IBM Corp., was used, adopting an alpha level of significance of 5%.

## 3. Results

**Figure 1:**
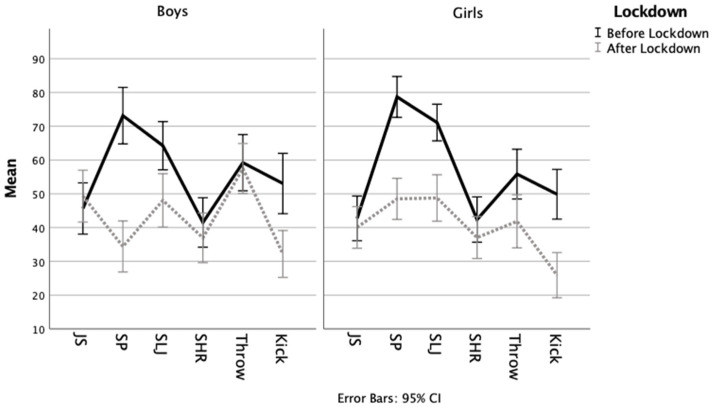
Means of motor component tests before and after lockdown. JS—jumping sideways; SP—shifting platforms; SLJ—standing long jump; SHR—shuttle run.

Our results indicate that, regardless of sex, motor performances after lockdown in five of the six tests (except for jumping sideways in boys) were lower than before lockdown (Table 1 and Figure 1).

As depicted in Table 1, there was a marked trend for children to shift from an upper to a lower quartile. There was a significant difference between the results before and after lockdown for the tests shifting platforms, standing long jump (both *p* < 0.001), and kicking velocity (*p* = 0.009) in boys. For example, on the shifting platforms test, 71.4% of children who previously belonged to the 3rd quartile, shifted to the 1st quartile after lockdown. A similar trend was observed in girls regarding the results before and after lockdown. For girls, in addition to the tests mentioned above (all *p* < 0.001) throwing velocity also showed a significant difference between the two moments (*p* = 0.041).

As shown in Table 2, there was a main effect of lockdown, with the results after lockdown being inferior to the results before lockdown (*p* values between 0.007 to <0.001) in all motor tests (except jumping sideways), in the three components of MC, and in global MC. Additionally, the results on the shifting platforms test showed a main effect of sex, since girls outperformed boys in that task. Lastly, on the throwing velocity test, there was a sex effect and an interaction effect between sex and lockdown, indicating that boys had a higher performance in this task and that the decrease in performance after the lockdown was more pronounced in girls.

## 4. Discussion

The results of this study confirmed the hypothesis that the imposed movement restrictions had a negative effect on children’s MC. In fact, a consistent decreasing trend was found in global MC, in its components and in individual tests (except jumping sideways), after lockdown when compared to the results before lockdown. The fact that no major differences between boys and girls were found was not surprising. As we could see in previous studies, PA decreased in all children alike, independently of their gender [23]. This fact is due probably because all children were confined to their homes. Usually in these ages, vigorous PA requires large spaces, and since the relationship between PA and MC is bidirectional [7,12,13], they were restrained to similar behavior.

Knowing that these type of restrictions are adverse to children’s physical activity behaviors [36,37] and that during lockdown the Portuguese children were less active, more sedentary, and more engaged in recreational screen-based activities than in the pre-lockdown period [23], we now confirm that this period was also deleterious to their MC.

When Stodden et al. [7] published their theoretical framework, it was hypothesized that PA promotes MC via a variety of exploratory as well as context-specific (i.e., structured activities, games, and sports) movement experiences. As a child ages, this relationship is hypothesized to become more reciprocal. Higher levels of MC foster more PA and, reciprocally, more PA fosters greater MC, which creates a positive spiral of engagement in PA across childhood and into adolescence [7]. If these movement experiences are (partly) excluded from children’s lives, it is expected that they cannot fully develop them. In that sense, we can postulate some reasons for our results. In Portugal, school programs were transferred to a mixed system of broadcast television and online homeschooling from April to June and all sports training activities were suspended until September 2020. School provides a fundamental environment to promote the development of MC for all children alike mostly due to two main reasons. Firstly, physical education (PE) holds the potential to enhance overall motor competence in children, as demonstrated in a recent meta-analysis [38]. In Portugal, PE is mandatory, from pre-school up to 12th grade. Time allocated to those classes ranges from 90 to 150 min/week over 2 or 3 sessions/week and is taught by a certified teacher [30]. In fact, the PE national curriculum is built in a progressive manner, aimed at the global and harmonious development of the child. Secondly, recess is generally viewed as a time for students to be active, so few restrictions to movement are imposed during that time [30]. The physical environment of the school has long been recognized as an effective setting for PA initiatives [39]. In fact, the combined lunchtime and recess PA has been reported to contribute to up to 40% of children’s recommended daily PA [40].

Another factor that probably contributed to these results is the fact that although quick walks and outdoor playtime (20 min) were permitted during the lockdown, children were encouraged to spend as little time as possible outdoors and to maintain social distance between themselves. A versatile outdoor environment can offer appropriate and timely challenges and plays an important role in children’s MC development [41]. Time spent outdoors is positively related to PA and negatively related to sedentary behavior in children aged 3 to 12 years [42]. Additionally, even though Portuguese children have low levels of independent mobility [43,44], this constitutes an important factor for the total daily PA, since the percentage of children playing outdoors with a weekly frequency is three or more times higher than the percentage of children engaged in sports practice [45].

The decrease in children’s MC levels was also probably influenced by the suspension of sports training activities from April until September. Organized sports have been shown to increase children’s PA [46,47], and frequent participation in them has been found to improve MC [48,49]. Portugal had a percentage of 61.8% of 6- to 9-year-old children practicing some form of organized sports at least once per week before lockdown [50], but now these numbers have decreased. In fact, from 2019/2020 to 2020/2121, 172,991 young federated athletes were lost. [51].

Knowing that summer break, can be a barrier to children’s engagement in positive health behaviors [52,53,54,55] and that gross motor skills before summer break significantly predict school PA after this break [56], the question that arises is what impact will the results of this study have in the future? Will children be able to catch up from this greater inactivity period? If yes, how long will it take and what strategies should we adopt to assure a fast recovery?

Considering that MC levels during childhood positively influence PA levels along the lifespan [9,11], and even though the long-term results of this lockdown period are yet to be determined, it is necessary to think about solutions to protect against sedentarism and minimize the immediate effect that the lockdown had on children’s MC.

To our knowledge, this is the first study to examine the impact of the COVID-19 lockdown on children’s MC. It is recommended that future research focus on the longitudinal observation of the MC and PA behaviors of today’s children to understand what kind of measures need to be taken to minimize the negative impact of lockdown.

## 5. Limitations and Future Directions

Although this study provides important information considering children’s MC after the lockdown, it is important to highlight that it has some limitations. First, we did not collect any data regarding other variables that were probably affected by the lockdown and that could have been studied, such as children’s body mass index, waist circumference, fitness level or physical activity habits. Some of these variables could have had moderating effects on motor competence that we were not able to investigate. Lastly, we did not collect any data on children’s socioeconomic status, which probably influenced the way families dealt with the lockdown and might also have impacted the results post-lockdown.

## 6. Conclusions

This study shows the objectively measured harmful impact of the lockdown on children’s MC. Children’s performance, assessed by a standardized MC test, the MCA [2], was significantly lower after the lockdown in all motor tests (except jumping sideways), in the three components of MC, and in global MC (an average of 13 points in boys and 16 points in girls), with most children shifting from an upper to a lower quartile between the pre- and post-lockdown periods. Even though the long-term impacts of this period are still unknown, the COVID-19 pandemic has disrupted many aspects of this generation’s lives, affecting PA and sedentary behaviors, screen time, eating habits, and even motor development. Future research should try to understand the evolution of MC and PA behaviors after this pandemic.

## Figures and Tables

**Table 1:** Cross-tabulation of the quartile distribution in all motor tests before and after lockdown, according to the MCA normative values, and McNemar–Bowker Test results.

			**Jumping Sideways AL**					**Shifting Platforms AL**			
			1st Q	2nd Q	3rd Q	4th Q		1st Q	2nd Q	3rd Q	4th Q
Boys	Jumping Sideways BL	1st Q	78.6%	14.3%	7.1%	0%	Shifting Platforms BL	100%	0%	0%	0%
		2nd Q	23.5%	13.5%	35.3%	17.6%		66.7%	33.3%	0%	0%
		3rd Q	0%	16.7%	41.7%	41.7%		71.4%	14.3%	14.3%	0%
		4th Q	0%	27.3%	27.3%	45.5%		31.4%	25.7%	28.6%	14.3%
			T_MB_ (5) = 4.167, *p* = 0.526					T_MB_ (6) = 40.000, *p* < 0.001			
Girls	Jumping Sideways BL	1st Q	63.6%	36.4%	0%	0%	Shifting Platforms BL	66.7%	33.3%	0%	0%
		2nd Q	15.0%	33.3%	33.3%	8.3%		42.9%	42.9%	14.3%	0%
		3rd Q	11.8%	41.2%	47.1%	0%		12.5%	6.,5%	12.5%	12.5%
		4th Q	0%	11.1%	44.4%	44.4%		4.8%	35.7%	42.9%	16.7%
			T_MB_ (5) = 9.091, *p* = 0.105					T_MB_ (6) = 36.877, *p* < 0.001			
			**Standing Long Jump AL**					**Shuttle Run AL**			
			1st Q	2nd Q	3rd Q	4th Q		1st Q	2nd Q	3rd Q	4th Q
Boys	Standing long Jump BL	1st Q	83.3%	16.7%	0%	0%	Shuttle Run BL	81.3%	18.7%	0%	0%
		2nd Q	44.4%	44.4%	0%	11.1%		42.1%	31.6%	26.3%	0%
		3rd Q	35.3%	23.5%	41.2%	0%		16.7%	41.7%	33.3%	8.3%
		4th Q	0%	13.6%	36.4%	50%		0%	14.3%	14.3%	71.4%
			T_MB_ (5) = 20.800, *p* = 0.001					T_MB_ (5) = 5.273, *p* = 0.384			
Girls	Standing long Jump BL	1st Q	100%	0%	0%	0%	Shuttle Run BL	58.8%	35.3%	5.9%	0%
		2nd Q	50.0%	30.0%	20.0%	8.3%		42.1%	47.4%	5.3%	5.3%
		3rd Q	11.8%	52.9%	23.5%	11.8%		6.3%	37.5%	50.0%	6.3%
		4th Q	9.4%%	29.4%	72.7%	80%		12.5%	0%	50.0%	37.5%
			T_MB_ (6) = 30.343, *p* < 0.001					T_MB_ (6) = 7.657, *p* = 0.264			
			**Throwing Velocity AL**					**Kicking Velocity AL**			
			1st Q	2nd Q	3rd Q	4th Q		1st Q	2nd Q	3rd Q	4th Q
Boys	Throwing Velocity BL	1st Q	33.3%	50.0%	8.3%	8.3%	Kicking Velocity BL	66.7%	26.7%	0%	6.7%
		2nd Q	12.5%	62.5%	0%	25.0%		44.4%	44.4%	0%	11.1%
		3rd Q	7.7%	46.2%	23.1%	23.1%		35.7%	35.7%	21.4%	7.1%
		4th Q	4.8%	4.8%	38.1%	52.4%		31.3%	31.3%	25.0%	12.5%
			T_MB_ (6) = 12.177, *p* = 0.058					T_MB_ (6) = 17.133, *p* = 0.009			
Girls	Throwing Velocity BL	1st Q	53.8%	15.4%	23.1%	7.7%	Kicking Velocity BL	81.3%	18.7%	0%	0%
		2nd Q	45.5%	0%	54.5%	0%		70.0%	10.0%	20.0%	5.3%
		3rd Q	52.9%	23.5%	17.6%	5.9%		34.8%	34.8%	13.0%	17.4%
		4th Q	26.3%	21.1%	21.1%	31.6%		63.6%	18.2%	18.2%	0%
			T_MB_ (6) = 13.152, *p* = 0.041					T_MB_ (6) = 2.867, *p* = 0.001			

BL—before lockdown; AL—after lockdown. T_MB_—McNemar–Bowker Test.

**Table 2:** Repeated measures ANOVA (sex * moment) for all motor tests, motor competence and respective categories.

	Boys (*N* = 54) Age M BL 7.49 ± 0.93 Age M AL 8.42 ± 0.90		Girls (*N* = 60) Age M BL 7.48 ± 0.86 Age M AL 8.38 ± 0.88		
	BL	AL	BL	AL	
	Mean ± SD	Mean ± SD	Mean ± SD	Mean ± SD	Repeated ANOVA
Jumping Sideways	45.64 ± 27.81	49.31 ± 28.13	42.72 ± 25.61	40.05 ± 23.85	F lockdown (1, 112) = 0.07, *p* = 0.794, ηp^2^ = 0.001 F sex (1, 112) = 1.78, *p* = 0.184, ηp^2^ = 0.016 F lockdown *sex (1, 112) = 2.81, *p* = 0.096, ηp^2^ = 0.025
Shifting Platforms	73.12 ± 30.65	34.40 ± 27.68	78.68 ± 23.40	48.51 ± 23.57	F lockdown (1, 112) =209.82 *p* < 0.001, ηp^2^ = 0.652 F sex (1, 112) = 5.15, *p* = 0.025, ηp^2^ = 0.044 F lockdown *sex (1, 112) = 3.24, *p* = 0.075, ηp^2^ = 0.028
Standing Long Jump	64.24 ± 26.13	48.04 ± 28.97	71.10 ± 20.98	50.15 ± 25.05	F lockdown (1, 112) =94.64, *p* < 0.001, ηp^2^ = 0.057 F sex (1, 112) = 1.06, *p* = 0.305, ηp^2^ = 0.007 F lockdown *sex (1, 112) = 1.54, *p* = 0.217, ηp^2^ = 0.021
Shuttle Run	41.52 ± 26.82	36.97 ± 26.94	42.37 ± 25.98	37.01 ± 25.33	F lockdown (1, 112) =7.51, *p* = 0.007, ηp^2^ = 0.063 F sex (1, 112) = 0.01, *p* = 0.918, ηp^2^ = 0.000 F lockdown *sex (1, 112) = 0.46, *p* = 0.830, ηp^2^ = 0.000
Throwing Velocity	59.21 ± 30.50	57.49 ± 27.08	55.83 ± 28.47	41.85 ± 30.40	F lockdown (1, 112) =6.71, *p* = 0.011, ηp^2^ = 0.057 F sex (1, 112) = 4.36, *p* = 0.039, ηp^2^ = 0.037 F lockdown *sex (1, 112) = 4.09, *p* = 0.046, ηp^2^ = 0.035
Kicking Velocity	53.04 ± 32.71	32.18 ± 25.47	49.86 ± 28.52	25.91 ± 25.90	F lockdown (1, 112) = 57.20, *p* < 0.001, ηp^2^ = 0.338 F sex (1, 112) = 1.16, *p* = 0.284, ηp^2^ = 0.010 F lockdown *sex (1, 112) = 0.27, *p* = 0.603, ηp^2^ = 0.002
Stability	59.39 ± 25.48	41.85 ± 23.68	60.70 ± 20.27	44.28 ± 20.43	F lockdown (1, 112) =129.19, *p* < 0.001, ηp^2^ = 0.536 F sex (1, 112) = 0.225, *p* = 0.636, ηp^2^ = 0.002 F lockdown *sex (1, 112) = 0.14, *p* = 0.71, ηp^2^ = 0.001
Locomotor	52.88 ± 23.74	42.50 ± 26.20	56.73 ± 20.82	43.60 ± 22.01	F lockdown (1, 112) =64.43, *p* < 0.001, ηp^2^ = 0.363 F sex (1, 112) = 0.36, *p* = 0.547, ηp^2^ = 0.002 F lockdown *sex (1, 112) = 0.89, *p* = 0.349, ηp^2^ = 0.011
Manipulative	56.13 ± 25.96	44.83 ± 21.96	52.85 ± 22.52	33.88 ± 21.46	F lockdown (1, 112) =58.20, *p* < 0.001, ηp^2^ = 0.342 F sex (1, 112) = 3.46, *p* = 0.066, ηp^2^ = 0.030 F lockdown *sex (1, 112) = 3.74, *p* = 0.056, ηp^2^ = 0.032
MC	56.13 ± 20.03	43.06 ± 19.66	56.76 ± 17.10	40.58 ± 17.12	F lockdown (1, 112) =172.80, *p* < 0.001, ηp^2^ = 0.603 F sex (1, 112) = 0.08, *p* = 0.778, ηp^2^ = 0.001 F lockdown *sex (1, 112) = 1.95, *p* = 0.165, ηp^2^ = 0.019

BL—before lockdown; AL—after lockdown; M—Mean; MC—Motor Competence.
